# Confocal photoluminescence investigation to identify basal stacking fault’s role in the optical properties of semi-polar InGaN/GaN lighting emitting diodes

**DOI:** 10.1038/s41598-019-46292-8

**Published:** 2019-07-05

**Authors:** Y. Zhang, R. M. Smith, L. Jiu, J. Bai, T. Wang

**Affiliations:** Department of Electronic and Electrical Engineering, University of Sheffield, Mappin Street, Sheffield, S1 3JD United Kingdom

**Keywords:** Inorganic LEDs, Nanophotonics and plasmonics

## Abstract

High spatial-resolution confocal photoluminescence (PL) measurements have been performed on a series of semi-polar (11–22) InGaN light emitting diodes (LEDs) with emission wavelengths up to yellow. These LED samples have been grown on our high crystal quality semi-polar GaN templates which feature periodically distributed basal stacking faults (BSFs), which facilitates the study of the influence of BSFs on their optical performance. Scanning confocal PL measurements have been performed across BSFs regions and BSF-free regions. For the blue LED, both the emission intensity and the emission wavelength exhibit a periodic behavior, matching the periodic distribution of BSFs. Furthermore, the BSF regions show a longer emission wavelength and a reduced emission intensity compared with the BSF-free regions. However, with increasing indium content, this periodic behavior in both emission intensity and emission wavelength becomes weaker and weaker. When the indium content (and correspondingly, wavelength) increases up to achieve yellow emission, only random *fluctuations have* been observed. It is worth highlighting that the influence of BSFs on the optical properties of semi-polar InGaN LEDs is different from the role of dislocations which normally act as non-radiative recombination centers.

## Introduction

Further development of III-nitride optoelectronics, predominantly based on *c-plane* substrates, is facing a number of great challenges as a result of polarization induced electrical fields, one of the fundamental limitations for current InGaN/GaN based visible emitters. This becomes more and more severe with increasing emission wavelength, thus generating the well-known “green/yellow gap”^1^. Furthermore, analogous to the evolution of conventional telephones to smartphones, “smart-lighting” needs to be featured with multiple functions, for example, emitters simultaneously used for general illumination and visible light communication (i.e., Li-Fi), where InGaN/GaN emitters with an ultra-fast response are the key components. However, current InGaN/GaN based emitters cannot meet the key requirements due to the polarization issue, leading to a very limited bandwidth which is typically on a 100 s of MHz scale^2^.

Compared with III-nitride optoelectronics on *c-plane* substrates, semi-polar GaN, in particular (11–22) GaN, is expected to exhibit a number of major advantages in fabricating InGaN based emitters with longer wavelengths beyond the blue spectral region, *e*.*g*. green and yellow, which are critical for solid-state lighting, Li-Fi and opto-genetics. Due to significantly reduced polarization, III-nitride emitters grown on semi-polar (11–22) GaN exhibit enhanced internal quantum efficiency (IQE) and significantly reduced radiative recombination lifetimes which are down to 100 s of picoseconds from 10 s of nanoseconds for *c-plane* III-nitride emitters^3–5^. Furthermore, InGaN grown on (11–22) GaN shows a significantly enhanced indium incorporation rate in comparison with those on either non-polar or polar surfaces^6^. As a result, (11–22) GaN is expected to be the most promising option for developing III-nitride emitters with longer wavelengths where high indium content is required. III-nitride visible emitters grown on (11–22) GaN also ideally meet the requirements for Li-Fi applications. Further information about the current status of the development of (11–22) GaN can be found in a topical review^7^.

However, it is crucial to obtain semi-polar (11–22) GaN with high crystal quality on industry-compatible and up-scalable substrates such as sapphire or silicon, which is extremely challenging. Recently, our group has achieved semi-polar (11–22) GaN on sapphire with significantly improved crystal quality by means of overgrowth on regularly-arrayed micro-rod templates^8,9^, leading to the demonstration of high-performance InGaN emitters in a wide spectral region of up to amber^10^.

It is well-known that dislocations are the main kind of defects in c-plane GaN on sapphire or silicon. In contrast, in addition to the dislocations present in conventionally heteroepitaxial c-plane GaN semi-polar GaN is affected by basal stacking faults (BSFs) which are mainly terminated by partial dislocations (PDs)^11–13^. Based on the previous experience on c-plane GaN on sapphire, it has been well understood that dislocations normally act as non-radiative recombination centers. In contrast, there are only a limited number of reports on investigating the influence of BSFs on optical properties. These studies are mainly on single semi-polar GaN films^14–17^, not InGaN.

Due to the great challenges in obtaining high crystal quality semi-polar GaN and InGaN with high indium content and also due to lack of a proper structure, there has been no systematic study on investigating the influence of BSFs on the optical properties of semi-polar InGaN quantum wells, in particular InGaN with high indium content. In this paper, we have employed both transmission electron microscopy (TEM) and confocal photoluminescence (PL) measurements (a spatial resolution of ~160 nm) to perform detailed investigations on a number of semi-polar (11–22) InGaN single quantum well (SQW) LEDs with different indium content in a wide spectral region from blue to yellow. These LED samples are grown on our semi-polar GaN templates with significantly improved crystal quality mentioned above. Furthermore, the BSFs distribute along the [−1–123] direction in a periodic manner, where the BSF-free regions are typically >2 µm wide and the width of the BSF regions is about 1 µm^18^. Such a configuration offers a unique opportunity to investigate the influence of BSFs on the optical properties of the InGaN SQW LEDs. Our results have demonstrated that for the semi-polar (11–22) blue LED the InGaN SQW featured with BSFs shows a redshift in emission wavelength and a slight reduction of emission intensity in comparison with the InGaN SQW free of BSFs. Furthermore, our results also indicate that BSFs play much less important role in the optical properties of the LEDs with higher indium contents. This is completely different from the influence of dislocations.

All the LEDs used for the present study were grown on our semi-polar GaN templates which were obtained by means of overgrowth on regularly arrayed micro-rod templates by metal organic chemical vapor deposition (MOCVD), where micro-rod arrays with a diameter of 4 µm and a micro-rod spacing of 4 µm (along the [−1–123] or the *m* direction) were employed for overgrowth. The dislocation density is around 2 × 10^8^/cm^2^ (which is lower than that of standard commercial c-plane counterparts) and the BSF density is around 1 × 10^4^/cm, *which have been obtained by TEM measurements*^18^. X-ray diffraction rocking curves as a function of azimuth angle show full-width-at-maxima (FWHM) of less than 360 arc sec and 252 arc sec at 0° and 90° azimuth angle, respectively^18^. This demonstrates an excellent crystal quality of our (11–22) GaN, approaching or being even better than current *c-plane* GaN on sapphire for growth of ultra-high brightness blue LEDs. Details of the fabrication of regularly arrayed micro-rod templates and the overgrowth have been reported elsewhere^8^. These overgrown semi-polar (11–22) SQW LEDs in each case consist of a 1 µm Si-doped GaN layer, a 3 nm InGaN well sandwiched in 9 nm GaN barriers, and a 150 nm Mg-doped GaN layer. *By means of employing a method introduced by Vickers et al*.^19^, *the indium composition has been determined to be from* 0.20 to 0.40 (labeled as Sample A, B, C, D, E, F in increasing indium content order), corresponding to an emission wavelength ranging from 458 to 571 nm at room temperature. Please refer to the supplemental material for their photoluminescence spectra measured at room temperature using a standard photoluminescence system, where a 375 nm diode laser is used as an excitation source.

*Confocal* PL measurements have been carried out at room temperature using a 375 nm continuous-wave diode laser as an excitation source. The lateral spatial resolution of our confocal microscopy system is around 160 nm, which is significantly smaller than the width of BSF regions (~1 µm) and the width of BSF-free regions (~2 µm) in these semi-polar SQW LEDs. Therefore, this spatial resolution is sufficient to investigate the difference in optical properties between the regions with and without BSFs. *Confocal* PL mapping has been measured by scanning areas of 80 × 80 µm^2^ on each LED sample.

**Figure 1:**
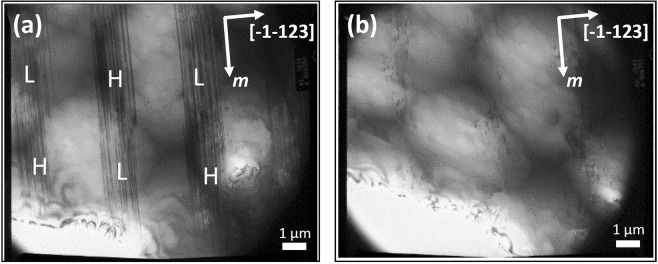
Bright-field plan-view TEM image of our semi-polar (11–22) overgrown GaN taken with a diffraction vector of ***g*** = 10–10 and ***g*** = 1–212 for (**a**,**b**), respectively. ‘H’ and ‘L’ label the areas with a high and a low BSF density, respectively.

As a standard microstructural characterization, Fig. 1a shows a plane-view TEM image of our high quality semi-polar GaN template taken along the [−1–120] zone axis with a diffraction vector of ***g*** = 10–10 which allows us to observe BSFs, indicating that BSFs distribute in a periodic manner, specifically, BSF regions are separated by BSF-free regions along the [−1–123] direction. Furthermore, in each BSF stripe region, the areas with a high or a low BSF density, labelled as ‘H’ and ‘L’ as shown in Fig. 1a, respectively, also distribute periodically along the *m* direction^18^. It has been reported that BSFs can penetrate through a quantum well region and then reach a sample surface with few extra BSFs generated at a GaN/InGaN heterointerface^20^. Consequently, such a periodic BSF distribution pattern can be transferred to the top surface from the underlying GaN layer. The appearance of BSF regions and BSF-free regions in a periodic manner offers us a unique opportunity to study the influence of BSFs on the optical properties of semi-polar (11–22) InGaN/GaN SQW LEDs.

Figure 1b shows a plan-view TEM image of our high quality semi-polar GaN template taken by tilting the semi-polar template specimen to the [22–43] zone axis with a diffraction vector of ***g*** = 1–212 which allows us to observe dislocations. The surface has been observed to be dislocation free in the BSF-free regions, while a small number of dislocations are only observed in the BSF regions.

**Figure 2:**
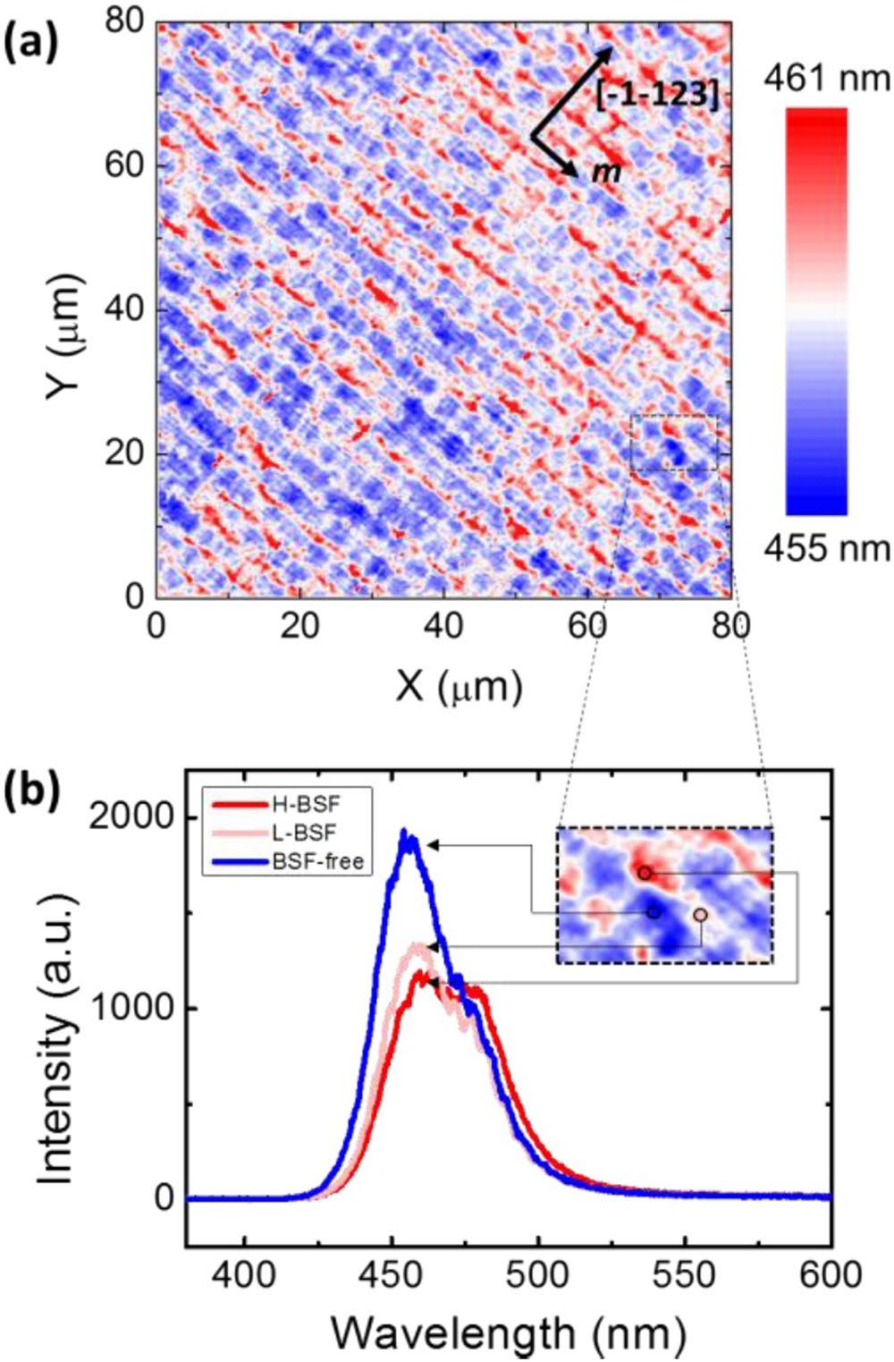
(**a**) Confocal PL mapping measured in an area of 80 × 80 µm^2^ from sample in terms of centre of mass emission wavelength; (**b**) Confocal PL spectra from three typical regions as denoted in inset.

Figure 2a presents a *confocal* PL emission wavelength map measured on **Sample A** at room temperature in an area of 80 × 80 µm^2^, exhibiting the areas (labeled as red color) representing a center of mass PL emission wavelength at ~461 nm and the areas (denoted as blue color) with a center of mass wavelength at 458 nm distributed in a periodic manner along the [−1–123] direction. Further examining Fig. 2a shows that the average widths of the areas (labeled as red color) and the areas (labeled as blue color) are around 1 and 2 µm, respectively, matching the BSF regions and the BSF-free regions as shown in Fig. 1a. Furthermore, on each “red” color region, there also exists periodic areas labeled as “pink” color that correspond to a medium center of mass PL emission wavelength at 459 nm. Such a wavelength distribution pattern is identical to the BSF distribution pattern as shown in Fig. 1a, indicating that the long and the short wavelength *emissions* come from the SQW with and without BSFs, respectively.

Figure 2b shows typical confocal PL spectra measured on the “blue color” region (i.e., BSF-free region), the “red color” region (i.e., BSF region with a high density of BSF density) and the “pink color” region (i.e., BSF region with a low density of BSF density) as shown inset. Figure 2b displays that the emission at 461 nm, 459 nm and 458 nm are from the areas with a high BSF density, the area with a low BSF density and the BSF-free region, respectively. The emission intensity from the BSF regions is approximately half that of those from the BSF-free regions, while the emission intensity from either a high BSF density or a low BSF density is similar. The small redshift in PL emission wavelength from the BSF regions compared with the BSF-free region is due to the nature of BSFs, as BSFs normally introduce a zinc-blende structure into wurtzite GaN^11,21^.

An extra shoulder on the low energy side (at 478 nm) from the areas (labeled as red color) with a high BSF density has been observed as shown Fig. 2b. This shoulder is attributed to slightly higher indium composition than those in the surrounding regions as a result of BSFs^22^. It is worth highlighting that the shoulder on the low energy side never appears in any BSF-free regions.

**Figure 3:**
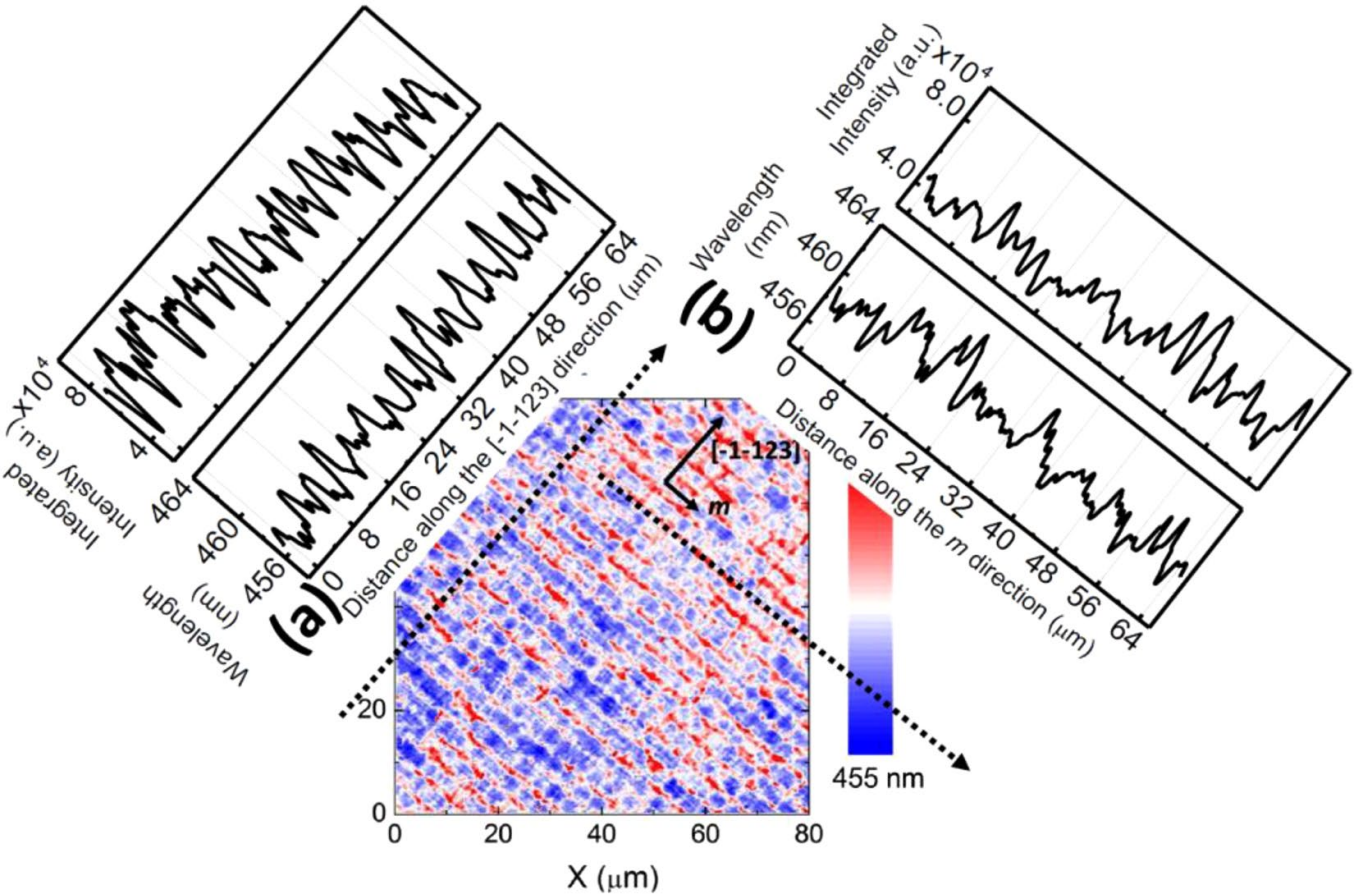
Profiles of centre of mass PL emission wavelength and PL emission intensity of the semi-polar (11–22) LED structure with a PL emission wavelength at 458 nm measured along the [−1–123]-direction (**a**); and along the m-direction (**b**).

Further evidence to support the above conclusion includes scanning *confocal* PL measurements carried out on **Sample A** along the [−1–123] direction and the *m* direction as schematically shown in Fig. 3.

Figure 3a shows a *confocal* PL map of **Sample A** measured along the [−1–123] (namely, PL scanning measurements through BSF regions and BSF-free regions periodically), exhibiting a periodic change in both emission wavelength and emission intensity. It is clearly demonstrated that BSF regions exhibit a long emission wavelength with a reduced intensity, while BSF-free regions show a short emission wavelength but with an enhanced intensity.

Figure 3b exhibits a *confocal* PL map of **Sample A** measured along the *m*-direction, showing a periodic change in emission wavelength, where the period is 8 µm which is consistent with the period of high or low BSF density regions as observed in our previous TEM measurements^18^. The PL intensity also exhibits fluctuation but with a less regular behavior compared with the emission wavelength behavior. Here, the weak correlation between PL intensity and BSF density further supports that BSFs might not play an important role in determining emission intensity. The reduction in emission intensity from the BSF regions compared with that from the BSF-free regions is due to the existence of dislocations acting as non-radiative recombination centers within the BSF regions (confirmed by Fig. 1b). These are partial dislocations which generally terminate BSFs when they reach an InGaN quantum well structure^23^. Furthermore, although the BSFs distribution is regular, the length of BSFs is random, making the distribution of partial dislocations distribute irregularly along the *m* direction. As a result, the dislocation distribution is less regular than those of BSFs along the *m* direction, leading to the weak correlation. Such *confocal* PL mapping measurements have been repeatedly performed on **Sample A** many times, showing a universe rule. This further confirms that the influence of BSFs on optical properties is different from that of dislocations which generally act as non-radiative recombination centres^24,25^.

**Figure 4:**
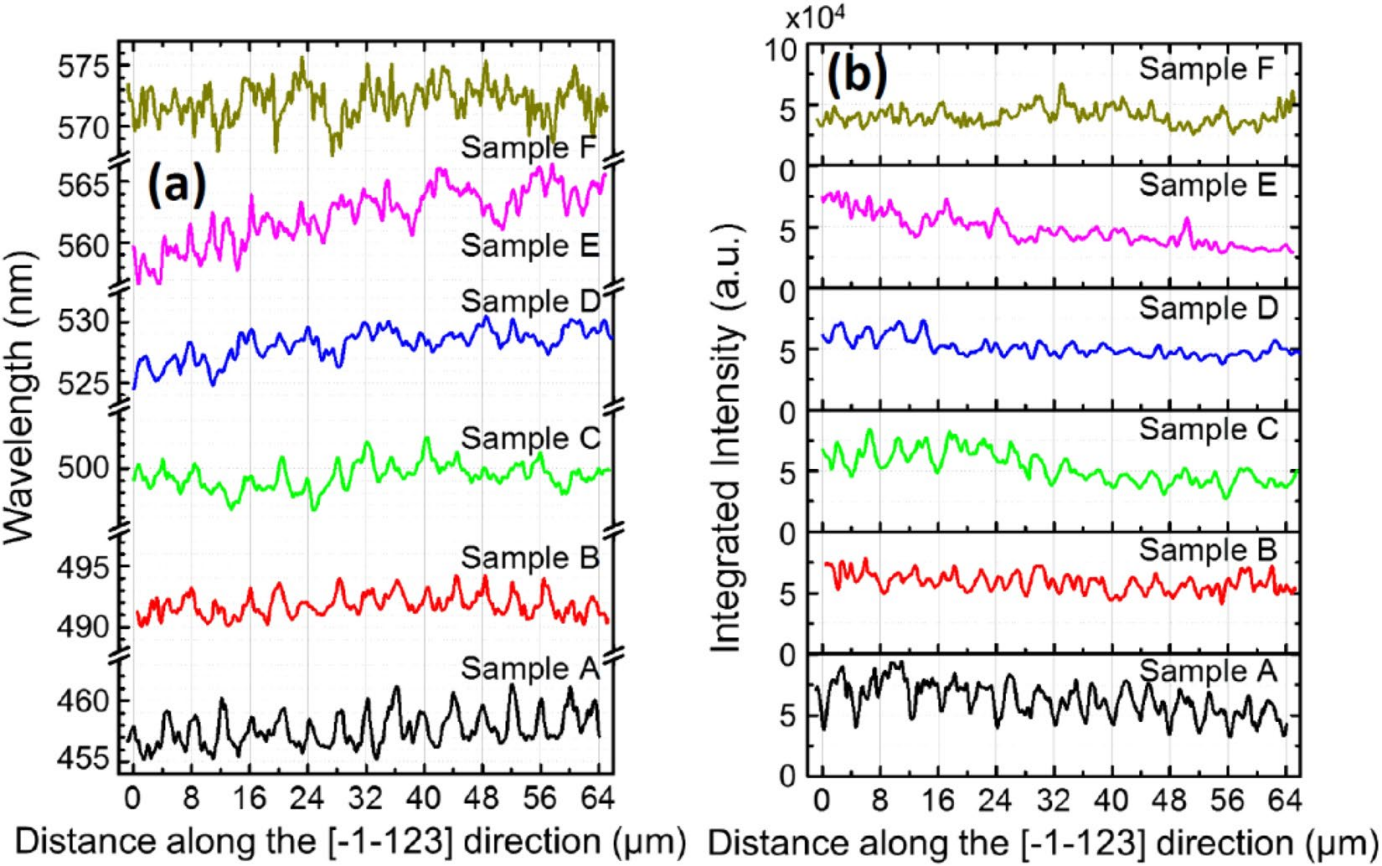
Profiles of centre of mass PL emission wavelength (**a**) and PL emission intensity (**b**) of all the LED structures as a function of indium content, measured along the [−1–123]-direction.

Further investigation has been carried out on Samples B, C, D, E and F with higher indium content of up to 0.4 (equivalent to 571 nm emission wavelength) in order to study the influence of BSFs on the optical properties of semi-polar InGaN SQW LED as a function of indium composition. Identical confocal PL mapping measurements to those on **Sample** A have been performed on the rest of the LED samples. Figure 4 presents the confocal PL mapping of all the LEDs as a function of indium content (i.e., as a function of emission wavelength). Figure 4a *displays* the profiles of all the LED samples in term of emission wavelength, while Fig. 4b shows the profiles in terms of emission intensity. All the confocal PL mapping has been measured by scanning along the [−1–123] direction, where BSF regions and BSF-free regions distribute in a periodic manner.

As shown in Fig. 4a, a clear periodic behavior has been observed for **Sample A** as we discussed above, indicating that a peak in center of mass PL emission wavelength profile corresponds to the emission from BSF regions, while a valley indicates the emission from BSF-free regions. With increasing indium content (i.e., emission wavelength) from 458 nm to 526 nm, the peak-to-valley wavelength variation decreases from ~4 nm to ~2 nm, meaning that the periodic behavior in emission wavelength become weak. This is due to an enhanced composition fluctuation as a result of increasing indium content, which makes the potential fluctuation within BSF regions be less and less pronounced. Therefore, the difference in emission wavelength between BSF regions and BSF-free regions reduces with increasing indium content. With further increasing emission wavelength to 571 nm, the emission wavelength profile exhibits more and more random fluctuations. It means that the fluctuation in emission wavelength as a result of natural indium segregation in InGaN becomes predominant in comparison with those induced by BSFs. For the emission intensity profile as shown in Fig. 4b, the same intensity range (0–10 × 10^4^ a.u.) is set for all the *y* axes. Apart from a reduction in emission intensity, the emission intensity fluctuation also exhibits more and more random with increasing indium composition. In a word, with increasing indium content, the periodic nature of both emission wavelength and emission intensity become weaker and weaker, and finally the distribution of both emission wavelength and emission intensity exhibits random behavior. Therefore, this demonstrates that BSFs do not play a critical role in the optical properties of semi-polar InGaN with high indium content.

In conclusion, the influence of BSFs on the optical properties of semi-polar (11–20) InGaN LEDs has been investigated as a function of indium content from blue to yellow by means of a high-resolution confocal PL mapping measurements. These LED samples have been grown on our semi-polar GaN templates with a low dislocation density and featured with BSFs distributing in a periodic manner along a specific direction which matches the configuration of the micro-rod arrayed templates used. Scanning confocal PL measurements have been performed across BSF regions and BSF-free regions periodically. For the blue LED, both the emission intensity and the emission wavelength exhibit a periodic behavior, which matches the periodic distribution of BSFs. However, with increasing indium content, this periodic behavior in both emission intensity and the emission wavelength becomes weaker and weaker, and finally they just *show random fluctuations* when the indium content increases to 0.4 (i.e., yellow emission at 571 nm).

## Methods

### Fabrication of regular microrod arrays

A single (11–22) GaN layer with a thickness of 0.4 μm is grown on our high temperature AlN buffer layer on *m-plane* sapphire substrate by metal organic chemical vapor deposition (MOCVD)^18^. Subsequently, a SiO_2_ film with a thickness of 500 nm as a mask is deposited on the as-grown semi-polar GaN using a standard plasma enhanced chemical vapour deposition (PECVD) technique. The SiO_2_ film is then selectively etched into regularly arrayed micro-rods with a diameter of 4 µm and a micro-rod spacing of 4 µm by means of employing a standard photolithography approach and then dry-etching processes. The regularly arrayed SiO_2_ micro-rod arrays are used as a second mask to finally etch the GaN layer underneath down to the sapphire substrate, forming regular semipolar GaN micro-rod arrays by standard dry-etching processes. Each SiO_2_ micro mask formed remains on top of each GaN micro-rod.

Confocal photoluminescence measurements have been carried out at room temperature using a 375 nm continuous-wave diode laser as an excitation source. The laser is focused on a sample by a 100× magnification objective with a 0.95 numerical aperture (NA). Luminescence from the sample is focused through a 10 µm pinhole. The lateral spatial resolution of our confocal microscopy system is around 160 nm.

## Supplementary information

Supplmentary information
