# Supplementary material for: Confocal photoluminescence investigation to identify basal stacking fault’s role in the optical properties of semi-polar InGaN/GaN lighting emitting diodes
Source: Sci Rep. 2019 Jul 5;9:9735. doi: 10.1038/s41598-019-46292-8 (PMC6611846; doi:10.1038/s41598-019-46292-8)
Supplement: Supplementary file 1 — Supplmentary information [file 41598_2019_46292_MOESM1_ESM.pdf]

Confocal photoluminescence investigation to identify basal stacking fault's role in the optical properties of semi-polar InGaN/GaN lighting emitting diodes

Y. Zhang[#], R. M. Smith[#], L. Jiu, J. Bai and T. Wang[*]

Department of Electronic and Electrical Engineering, University of Sheffield, Mappin Street, Sheffield, S1 3JD, United Kingdom

# Y. Zhang and R. M. Smith contributed equally to this work;

*E-mail: t.wang@sheffield.ac.uk

Supplementary material

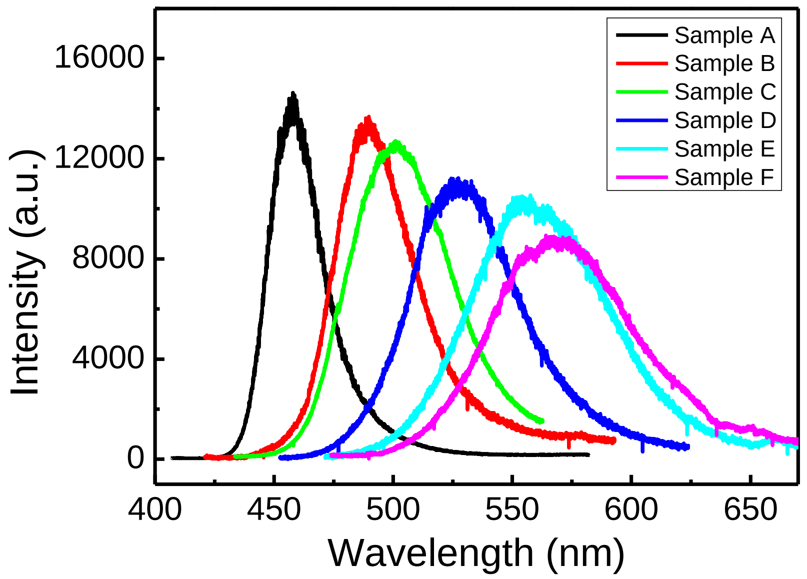

Fig. S1: Room temperature photoluminescence (PL) spectra of sample A-E using a 375 nm continuous-wave laser as an excitation source. The beam is focused on the sample with a spot diameter of about 0.2 mm, resulting in a power density of ~30 W/cm^2.
